# Suicide on Facebook-the tales of unnoticed departure in Bangladesh

**DOI:** 10.1017/gmh.2020.5

**Published:** 2020-05-26

**Authors:** Tanjir Rashid Soron, Sheikh Mohammed Shariful Islam

**Affiliations:** 1 Telepsychiatry Research and Innovation Network, Dhaka, Bangladesh; 2 University of Edinburgh, Edinburgh, UK; 3 Deakin University – Geelong Waterfront Campus, Geelong, Australia

**Keywords:** Bangladesh, Facebook, Post, Suicide, Livestream

## Abstract

Facebook has transformed social communication and offers the opportunity to share personal thoughts to people including suicide ideas, plans and attempts. Suicide after Facebook posts has been reported in different parts of the world and it has become a potential area of research for suicide prevention. The analysis of Facebook posts prior to suicide or Facebook live streaming may help in understanding the etiological factors, patterns of communication and possible prevention approaches for a particular community. However, there is a dearth of evidence about suicide incidents after Facebook posts and Facebook live streaming in low and middle-income countries. This study aims to explore the trends and phenomena of suicide after Facebook posts and live streaming in Bangladesh. We conducted an online search using the Google, Facebook and five daily online newspaper archives from 15th August to 15th September 2019. Two research assistants independently conducted the initial searching to find out people who committed suicide after Facebook posts or live streamed suicide in Bangladesh and documented 21 cases. After further evaluation of each of the 21 cases we confirmed 19 cases that met the selection criteria. All of them were under 35-years of age. We observed sucide after Facebook posts were more common in male(78%) e and students. Hanging was the most frequently used method of suicide followed by poisoning. Their Facebook posts and livestream videos indicated relationship problems, academic stress and mental disorders were the common stressors for their suicide. This study lays the foundation for the future researchers to work on suicidal posts on Facebook in Bangladesh and develop culture-specific, real-time suicide preventive systems using a social media platform.

## Introduction

Suicide in a hidden public health emergency causing an estimated 800000 deaths globally per year with about 85% occurring in low-and-middle income countries. The actual number of suicides might be much higher than this number as it is often under-reported due to multiple sociocultural, economic and political reasons (Patton *et al*., 2009 and Soron, 2018*a*). Patel and colleagues reported that the National Crime Records Bureau of India under-estimated suicides in men and women by at least 25 and 36% (Patel *et al*., 2012). The wide spread and increasing use of internet is helping us to collect more precise and accurate suicide data and the existing evidence shows a mixed relationship between internet use and suicide (Sedgwick *et al*., 2019). Internet potentiates the risk of suicide by providing access to various pro-suicide sites; at the same time, it helps to prevent suicide by offering easy access to supportive information and resources. Moreover, social Media has added a new dimension on the internet and provides novel opportunities for suicide prevention (Pauwels *et al*., 2017). Kaplan and Haenlein (2010) defined social media as ‘a group of Internet-based applications that build on the ideological and technological foundations of Web 2.0, and that allow the creation and exchange of User Generated Content’. Social media such as Facebook, Twitter, Weibo, Instagram, WhatsApp, Snapchat, YouTube, Google Plus, Blogs, Viber and MySpace has connected billions of people and provided the freedom of sharing thoughts, ideas and activities. However, different social networking sites offer different features in terms of facilities of interaction, their media offering and response time. The opportunity of using text, image, and video from a single social media platform made Facebook the most popular social media. Moreover, Facebook has less constraint on a post length than Twitter making Facebook more expressive. Facebook allow to express suicidal thoughts and attempts publicly (Young and Garett, 2018). Ruder *et al*. (2011) reported one of the first cases of committing suicide after expressing the suicidal idea on social media. Afterward, Luxton *et al*. (2012), Ma *et al*. (2016), Memon *et al*. (2018) and Soron (2019) reported multiple cases around the world where a person committed suicide after posting their intention on social media. This increasing tendency of committing suicide after social media posts or live-streamed suicide on social media poses new challenges for public health and safety. Simultaneously, it can provide the opportunity to reach the vulnerable groups early and provide interventions. Researchers and companies are now using the enormous interactions and behaviours on social media platforms to prevent suicide by mining social media posts and analysing the behaviours (Orooji and Langarizadeh, 2017).

People commit suicide for different reasons such as mental disorders (Mullins *et al*., 2019) povertyand other strains (Zhang, 2019), using different methods such as hanging (Callanan and Davis, 2012). Moreover, their pattern of suicide related social media posts and interaction varies based on the sociocultural and legal factors. Suicide is stigmatised and a criminal offense in Bangladesh that makes people uncomfortable in discussing suicide events in the country (Soron, 2019). However, suicide is frequently reported in details in the daily newspapers and people also share suicide of a friend or family member on their social media (Soron, 2018*a*). Moreover, people also express their suicidal ideas on Facebook before committing suicide and livestream the event on Facebook (Soron, 2019). Currently there is no evidence on the patterns of committing suicide after Facebook posts in Bangladesh. Therefore, we conducted this study to explore the trends and phenomenon of suicide after Facebook posts and live streaming in Bangladesh.

## Methods

We discussed with local mental health experts to determine the methodology for collecting information on pattern of suicide after Facebook posts or live streamed suicide cases. An exploratory search was conducted to finalise the Google search themes/keywords related to suicide in Bangladesh. The online search was performed using the keywords ‘Suicide after Facebook Post in Bangladesh’ and ‘Committing Suicide on Facebook Live in Bangladesh’ using Google Search Engine, five online newspapers (three Bangla and two English Daily Newspapers) and Facebook. No age, sex or time limitations were imposed.

The inclusion criteria were at least two different sources documenting a case of suicide after at least a Facebook post describing his/her intention to commit suicide or it was live-streamed on Facebook. We followed three steps filtering to confirm the cases. The initial search was conducted from August 15th to 30th of 2019. Two volunteers independently searched for the cases and documented the findings in a Microsoft Excel Sheet ensuring its confidentiality and other ethical issues. The lead researcher reviewed the details of the initial cases and selected the final cases that met the inclusion criteria.

## Results

A total of 21 cases were identified, of which 19 cases were included in this study which met the inclusion criteria. Sixteen people committed suicide after sharing their suicidal intention on Facebook and three persons live-streamed their suicide on Facebook. All deceaseds were under 35 years of age at the time of their suicide. Males committed suicide 4 times more frequently after Facebook posts. About 74% of the cases were students and others were media workers, physicians and musicians. Most of the students were studying at the University of Dhaka. About 70% of the suicide took place in the capital city Dhaka. However, suicide incidents were also reported from Chittagong, Sylhet, Comilla and Jessore.

Three of the deceaseds shared a series of posts mentioning their specific suicidal ideas several times before committing suicide. A Dhaka University student shared his last Facebook status from the rail line ‘I am going to commit a crime on my family, against the law & order. I am lying on a rail line. The train is coming. …………. …… I am leaving. Leaving useless myself forever’. He posted several similar depressing Facebook posts with a desire to die ‘You must have some quality to live unless you have to leave’, in another earlier post he wrote, ‘At last I realize that I am USELESS’. Another student, a media worker also committed suicide after a series of posts.

About 16% of the deceased mentioned tentative date of suicide. For example, a media worker posted on her Facebook ‘I will commit suicide tomorrow………’. However, all of the people committed suicide after the tentative date. These posts might have been their ultimate threat or desperate cry for help. Participants waited even after the deadlines for solutions or support for help. Hanging was the most common method of committing suicide (about 58%) followed by taking of lethal dose of drugs and jumping from a high-rise building. An unusual method of suicide ‘taking lethal dose of insulin’ by intravenous injectable form was also documented.

The common stressors of suicides as mentioned by the participants included academic pressure, relationship problem and mental disorders, specifically depression. The failure to cope with these stresses provoked them to commit suicide. A student at Dhaka University posted on his Facebook ‘Our education system has told us what to do and what not to … …I want freedom… even if it kills me’. A 23-year student who was suffering from mental illness wrote ‘I am choosing to do this because I've lost my track of life long ago. I tried a lot to get back on it, but I just can't break the loop’. Another person wrote being disappointment from his relation ‘There is no punishment for cheating in our country so I'm taking matters into my own hands and choosing the path of eternal peace’. Facebook friends and followers of the participants responded differently on a suicidal Facebook posts depending on the persona of the person, relationship with the participants and perceptions of the seriousness of the post. In most instances, friends and followers were sympathetic. However, in three cases there was evidence of mocking and provoking comments and funny feedbacks with emoji.

## Discussion

This is the first study in Bangladesh to report suicide cases after Facebook posts or live-streamed on Facebook and determine the common factors associated with suicide. World Health Organization Global Health Observatory reported suicide rate in Bangladesh 7.8 per 100000 population per year and Mashreky *et al*. (2013]) considered suicide cost more than 10000 live every year in the country that indicated more than 40 000 suicide occurred in Bangladesh from 2016 to 2019. However, there is a wide difference among the different sources (Soron, 2018*b*). In this study, we found 19 people committed suicide either after a post on Facebook expressing their intention to commit suicide or they committed the suicide live. However, the number of suicides after Facebook post is increasing and students were the most vulnerable group. Academic stress and relationship problems were frequently mentioned as the key provoking factors. Four deceaseds used their Facebook posts and Facebook live statements to protest different personal issues such as betrayal of partners, social issues such as against academic systems. Similar findings were reported in the different part of the world (Bock, 2019; Student Poverty, 2019; Pauline Dad Live Streams, 2017).

Though suicide is one of the leading causes of global mortality, there is no silver bullet to prevent it. A holistic, collaborative and culture-specific approach may reduce the burden of suicide. Social media opens a new horizon for research and real-time service development. Various mental health condition and disorders including major depressive disorder (Chung and Pennebaker, 2007; De Choudhury *et al*., 2013), post-traumatic stress disorder (Coppersmith et al., 2014*b*; Cohan *et al*., 2016), schizophrenia (Mitchell *et al*., 2015), eating disorders (Walker *et al*., 2015; Chancellor *et al*., 2016), generalised anxiety disorder, bipolar disorder (Coppersmith et al., 2014*a*), self-harm (Yates *et al*., 2017) and suicide (Coppersmith *et al*., 2015; Kumar *et al*., 2015) can be detected early following social media posts and behaviours analysis of the users (Aladağ *et al*., 2018; Coppersmith *et al*., 2018; Intahchomphoo, 2018; Brown *et al*., 2019). People share different posts for different motives on social media. Sometimes the same Facebook post carries different meaning to different people and people share fake news or fabricated information to draw the attention of friends, family or the community. This tendency was also observed in Bangladesh, for example people used Facebook as a convenient and affordable medium to protest and draw attention Poell (2018). Moreover, the discussion of suicide and reliability of data related to suicide depends on the political and legal attitude of a particular community and country.

According to the Bangladesh Law, an attempt of suicide and abetment of suicide is a criminal offense (The Penal Code, 1860). People are afraid of sharing their suicidal ideas and attempts due to this law and fear of punishment (Soron, 2019). However, they were comfortable expressing their suicidal thoughts and plans on social media or live-streaming the suicides on Facebook. The restricted access of sharing the distress and help seeking from the existing health care and social systems might influence suicide in Bangladesh. People may consider Facebook as an easily available platform to share their suicidal thoughts and plans to get the attention and help from the community. However, Facebook friends and followers are not always helpful and supportive, sometimes they may provoke them to commit suicide with critical comments or harsh emoji (Orooji and Langarizadeh, 2017; Soron, 2019). It is necessary to promote awareness among Facebook users to take Facebook suicide-related posts seriously and how people should respond to these suicide-related posts on Facebook. Suicide among students is increasing at an alarming rate in Bangladesh that needs a holistic approach by increasing awareness, access to mental health services including digital health initiatives and supportive educational systems. A culture-specific national guideline and its successful implementation may reduce the overall suicide rate. The increasing active Facebook users in Bangladesh may be used to prevent suicide. Facebook is developing a suicide database for Bangladesh (Soron, 2018*b*). Social media can help to increase access to care by leveraging help-seeking barriers and enable ambivalent individuals to access the mental healthcare system (Notredame *et al*., 2018)..Facebook uses artificial intelligence-based system which could be used to detect suicide risk and notify healthcare authorities and police to take preventive measures. The automatic detection of suicidal thoughts and behaviours and preventive measures may be helpful to prevent future suicide cases (Liu *et al*., 2019). Police helped people who were at risk of suicide after receiving an alert call from Facebook (Singer, 2018). The use of artificial intelligence and machine learning has received increased attention in recent days for suicide prediction (Fonseka et al., 2019). In addition to social media data, the digital assistant product makers such as Apple (Siri), Google Assistant and Amazon (Echo/Alexa) are augmenting the suicide prevention services by analysing to an indication of suicidal thoughts and behaviours (De *et al*., 2018). Future research into the use of Facebook algorithms for predicting suicide risks and alerting relevant authorities in developing countries are warranted to reduce unwanted and premature deaths which are mostly preventable.

## Conclusion

This is the first case series on Facebook and suicide in Bangladesh that highlights few key aspects of social media and suicide research. Further research on using social media to prevent suicide in Bangladesh is needed.
